# Supplementary material for: Optimization of SPIO Injection for Sentinel Lymph Node Dissection in a Rat Model
Source: Cancers (Basel). 2021 Oct 8;13(19):5031. doi: 10.3390/cancers13195031 (PMC8508039; doi:10.3390/cancers13195031)
Supplement: Supplementary file 1 [file cancers-13-05031-s001.zip › Supplementary_files_revision/Supplementary Data S1.pdf]

NOTE: Please save this file locally before filling in the table, DO NOT work on the file within your internet browser as changes will not be saved. Adobe Acrobat Reader (available free here) is recommended for completion.

The ARRIVE guidelines 2.0: author checklist

The ARRIVE Essential 10

These items are the basic minimum to include in a manuscript. Without this information, readers and reviewers cannot assess the reliability of the findings.

Item		Recommendation	Section/line number, or reason for not reporting
**Study design**	1	For each experiment, provide brief details of study design including:	
		a. The groups being compared, including control groups. If no control group has been used, the rationale should be stated.	Lines 84-105, 123-135, 145-153
		b. The experimental unit (e.g. a single animal, litter, or cage of animals).	Lines 84-105, 123-135, 145-153
**Sample size**	2	a. Specify the exact number of experimental units allocated to each group, and the total number in each experiment. Also indicate the total number of animals used.	Lines 84-105, 123-135, 145-153, Figure S1
		b. Explain how the sample size was decided. Provide details of any *a priori* sample size calculation, if done.	Lines 78-80
**Inclusion and exclusion criteria**	3	a. Describe any criteria used for including and excluding animals (or experimental units) during the experiment, and data points during the analysis. Specify if these criteria were established *a priori*. If no criteria were set, state this explicitly.	Line 200
		b. For each experimental group, report any animals, experimental units or data points not included in the analysis and explain why. If there were no exclusions, state so.	Line 200
		c. For each analysis, report the exact value of *n* in each experimental group.	Lines 84-105, 123-135, 145-153, Figure S1
**Randomisation**	4	a. State whether randomisation was used to allocate experimental units to control and treatment groups. If done, provide the method used to generate the randomisation sequence.	Lines 80-82
		b. Describe the strategy used to minimise potential confounders such as the order of treatments and measurements, or animal/cage location. If confounders were not controlled, state this explicitly.	Lines 80-82
**Blinding**	5	Describe who was aware of the group allocation at the different stages of the experiment (during the allocation, the conduct of the experiment, the outcome assessment, and the data analysis).	N/A
**Outcome measures**	6	a. Clearly define all outcome measures assessed (e.g. cell death, molecular markers, or behavioural changes).	Lines 83-175
		b. For hypothesis-testing studies, specify the primary outcome measure, i.e. the outcome measure that was used to determine the sample size.	N/A
**Statistical methods**	7	a. Provide details of the statistical methods used for each analysis, including software used.	Lines 189-200
		b. Describe any methods used to assess whether the data met the assumptions of the statistical approach, and what was done if the assumptions were not met.	Lines 189-200, 273-295
**Experimental animals**	8	a. Provide species-appropriate details of the animals used, including species, strain and substrain, sex, age or developmental stage, and, if relevant, weight.	Lines 80-82
		b. Provide further relevant information on the provenance of animals, health/immune status, genetic modification status, genotype, and any previous procedures.	N/A
**Experimental procedures**	9	For each experimental group, including controls, describe the procedures in enough detail to allow others to replicate them, including:	
		a. What was done, how it was done and what was used.	Materials and Methods section
		b. When and how often.	Materials and Methods section
		c. Where (including detail of any acclimatisation periods).	Materials and Methods section
		d. Why (provide rationale for procedures).	Materials and Methods section
**Results**	10	For each experiment conducted, including independent replications, report:	
		a. Summary/descriptive statistics for each experimental group, with a measure of variability where applicable (e.g. mean and SD, or median and range).	Results section, Tables S1 and S2
		b. If applicable, the effect size with a confidence interval.	Results section, Tables S3 and S4
