# Supplementary material for: Optimization of SPIO Injection for Sentinel Lymph Node Dissection in a Rat Model
Source: Cancers (Basel). 2021 Oct 8;13(19):5031. doi: 10.3390/cancers13195031 (PMC8508039; doi:10.3390/cancers13195031)
Supplement: Supplementary file 1 [file cancers-13-05031-s001.zip › Supplementary_files_revision/Supplementary_Tables.pdf]

Supplementary tables

Optimization of SPIO injection for sentinel lymph node dissection in a rat model.

Mirjam C.L. Peek†1, Kohei Saeki†*2§, Kaichi Ohashi3, Shinichi Chikaki3, Rose Baker4, Takayuki Nakagawa2, Moriaki Kusakabe5, Michael Douek1 and Masaki Sekino3.

1 Division of Cancer Studies, King's College London, Guy's Hospital Campus, Great Maze Pond, London, UK

2 Laboratory of Veterinary Surgery, Graduate School of Agricultural and Life Sciences, University of Tokyo, Tokyo, Japan

3 Department of Electrical Engineering and Information Systems, Graduate School of Engineering, University of Tokyo, Tokyo, Japan

4 Department of Statistics, School of Business 612, Maxwell Building, University of Salford, Salford, UK

5 Advanced Technology Research Laboratory, Research Centre for Food Safety, Graduate School of Agri-cultural and Life Sciences, University of Tokyo, Tokyo, Japan

† These authors contributed equally to the work.

* Correspondence: ko-saeki@vet.ous.ac.jp (K.S.)

§ Current affiliation: Department of Veterinary Medicine, Okayama University of Science, Ehime, Japan

Table S1. Descriptive statistics of the dose increase experiment and the MRI experiment.

Group	Iron in the SLN (µg)	Iron at the injection site (µg)	Iron in the secondary node (µg)	Artefact size at the SLN on MRI (cm)
2	0.05 (0.04 , 0.09)	6.97 (4.47 , 7.58)	-	0 (0 , 0.14)
10	0.86 (0.32 , 1)	36.04 (25.33 , 159.89)	-	-
20	2.05 (1.71 , 3.06)	18.81 (17.92 , 32.63)	-	0.27 (0.2 , 0.29)
40	3.34 (2.6 , 4.49)	29.57 (25.79 , 30.63)	-	0.35 (0.27 , 0.44)
100	9.35 (6.29 , 15.55)	67.09 (43.86 , 76.38)	-	0.6 (0.49 , 0.63)
200	19.96 (13.49 , 24.48)	128.54 (111.24 , 179.38)	-	0.61 (0.56 , 0.7)
1000	64.93 (48.63 , 85.49)	589.74 (551.1 , 679.1)	40.79 (36.83 , 44.74)	-
2000	80.54 (57.9 , 139)	1205.05 (1101.51 , 1594.41)	68.59 (55.17 , 94.59)	0.86 (0.79 , 0.98)
2790	73.07 (49.12 , 98.5)	1716.23 (1497.99 , 1950.29)	73.96 (50.77 , 94.04)	-
4000	80.52 (61.26 , 98.01)	3052.68 (2627.55 , 3312.71)	-	-

Values were reported as median (range). SLN: sentinel lymph node. MRI; magnetic resonance imaging.

Table S2. Descriptive statistics of the dilution and time-course experiment.

Group		Iron in the SLN (μg)	Iron at the injection site (μg)	Iron in the secondary node (μg)
Dilution	Time (min)			
x1	10	0.46 (0.21 , 3.2)	185.72 (162.91 , 212.36)	-
	30	1.95 (0.47 , 5.01)	175.33 (134.95 , 192.14)	-
	60	1.6 (0.84 , 2.36)	188.22 (180 , 197.65)	-
	360	10.37 (7.14 , 13.24)	140.09 (62.65 , 187.89)	-
	1440	16.41 (15.86 , 21.26)	143.65 (139.44 , 151.64)	3.07 (2.82 , 3.32)
x2	10	2.17 (0.28 , 4.97)	186.83 (177.74 , 218.57)	-
	30	0.44 (0.36 , 2.45)	182.59 (162.5 , 209.01)	-
	60	2.6 (1.41 , 8)	167.99 (152.5 , 193.34)	-
	360	11.65 (9.9 , 13.29)	141.68 (97.93 , 181.13)	-
	1440	19.67 (14.82 , 28.62)	147.93 (112.19 , 158.87)	3.73 (2.62 , 4.85)
x5	10	0.66 (0.56 , 3.91)	194.7 (174.69 , 209.98)	-
	30	1.19 (0.48 , 2.48)	186.15 (118.13 , 209.8)	-
	60	3.79 (2.09 , 8.71)	169.85 (167.01 , 192.64)	-
	360	16.22 (12.74 , 17.32)	178.83 (120.37 , 189.29)	-
	1440	22.84 (16.51 , 31.67)	156.73 (146.59 , 169.79)	2.89 (0.76 , 5.02)
x10	10	3 (1.49 , 6.92)	169.06 (165.76 , 177.37)	-
	30	5.03 (1.13 , 8.13)	182.72 (168.93 , 195.74)	-
	60	2.68 (1.69 , 19.23)	176.3 (155.17 , 181.59)	-
	360	17.61 (14.73 , 20.38)	164.48 (160.96 , 171.18)	-
	1440	17.94 (13.89 , 23.3)	160.62 (157.31 , 161.72)	2.69 (0.92 , 4.46)

Values were reported as median (range). SLN: sentinel lymph node.

Table S3. Results of the regression analysis for square root of the SLN iron amount.

Variable	Value	Standard error	Z score	P value	95% Confidence intervals
constant	-1.24E+00	1.34E+00	-9.21E-01	0.3569	-3.873774, 1.396580
standard deviation sigma	5.85E-01	4.62E-02	1.26E+01	0.0000	0.494063, 0.675250
Time (min)	7.94E-03	7.66E-04	1.04E+01	0.0000	0.006439, 0.009440
Time sq	-4.09E-06	4.96E-07	-8.25E+00	0.0000	-0.000005, -0.000003
Dilution	1.56E-01	9.23E-02	1.68E+00	0.0926	-0.025785, 0.337471
Dilution sq	-7.75E-03	8.10E-03	-9.58E-01	0.3382	-0.023624, 0.008116
Injection site	3.46E-02	1.72E-02	2.01E+00	0.0445	0.000849, 0.068358
Injection site sq	-1.28E-04	5.55E-05	-2.31E+00	0.0208	-0.000237, -0.000019

Table S4. Results of the regression analysis for the iron amount at the injection site.

Variable	Value	Standard error	Z score	P value	95% Confidence intervals
constant	1.75E+02	7.20E+00	2.43E+01	0.000	160.890852, 189.105540
standard deviation sigma	2.19E+01	1.73E+00	1.26E+01	0.000	18.476101, 25.251812
Time (min)	-1.14E-01	2.56E-02	-4.44E+00	0.000	-0.164159, -0.063656
Time sq	6.33E-05	1.71E-05	3.70E+00	0.000	0.000030, 0.000097
Dilution	4.33E+00	3.43E+00	1.26E+00	0.207	-2.397195, 11.054380
Dilution sq	-3.28E-01	3.00E-01	-1.09E+00	0.275	-0.915252, 0.260188
