# Supplementary figures and images for: Optimization of SPIO Injection for Sentinel Lymph Node Dissection in a Rat Model
Source: Cancers (Basel). 2021 Oct 8;13(19):5031. doi: 10.3390/cancers13195031 (PMC8508039; doi:10.3390/cancers13195031)

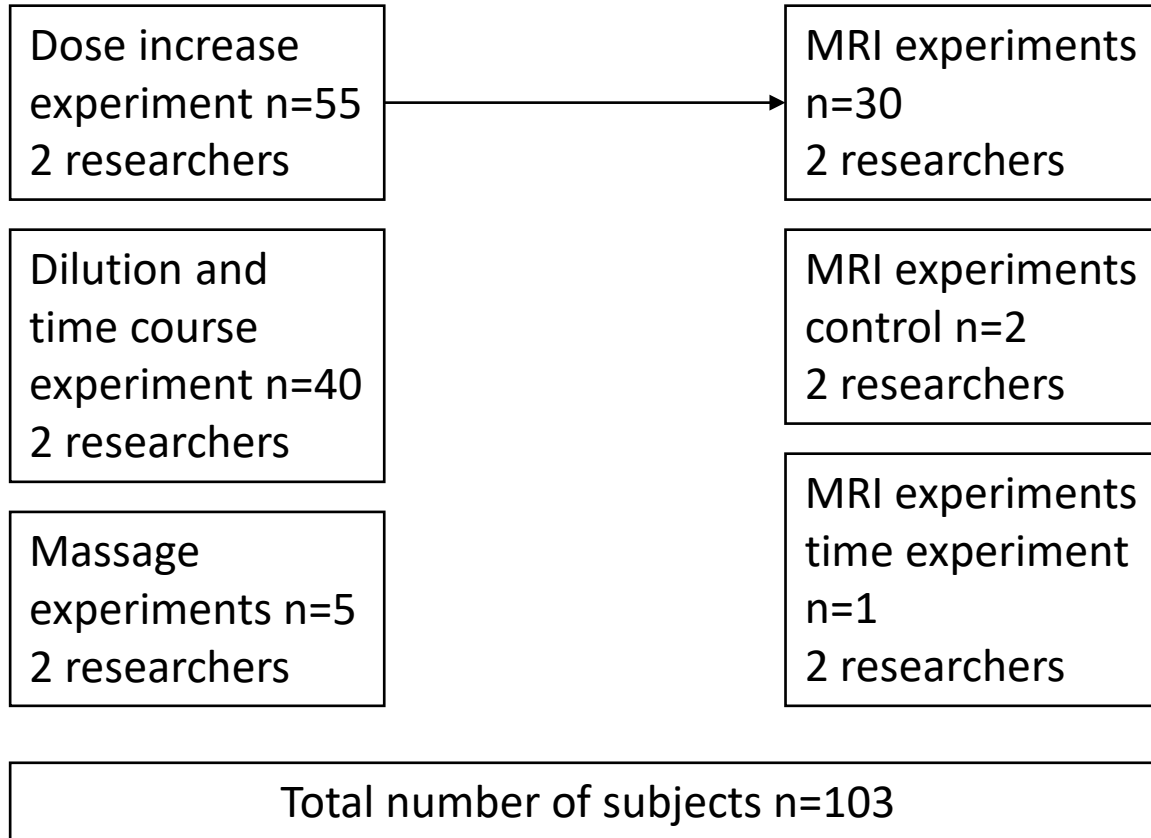

Supplement: Supplementary file 1 [file cancers-13-05031-s001.zip › Supplementary_files_revision/Figure S1.pdf]
